# Treatment of Relapsed B/T-cell Mixed Phenotype Acute Leukemia With Blinatumomab

**DOI:** 10.7759/cureus.40661

**Published:** 2023-06-19

**Authors:** Yasmeen Abdo, Geoffrey D Gibson, Sarika P Jain, Carter P Milner, Talal Hilal

**Affiliations:** 1 School of Medicine, University of Mississippi Medical Center, Jackson, USA; 2 Division of Hematology, University of Mississippi Medical Center, Jackson, USA; 3 Division of Pathology, University of Mississippi Medical Center, Jackson, USA; 4 Division of Hematology and Medical Oncology, Mayo Clinic, Phoenix, USA

**Keywords:** clinical hematology, mixed phenotype acute leukemia, b/t mixed acute leukemia, blinatumomab, bispecific t cell engager

## Abstract

Here, we describe the treatment of a patient with relapsed/refractory B/T mixed phenotype acute leukemia (MPAL) using blinatumomab monotherapy, the first bispecific T cell engager (BiTE) approved by the FDA for relapsed/refractory B cell acute lymphoblastic leukemia (B-ALL). A 64-year-old man with a history of stage 3 chronic kidney disease and type 2 diabetes mellitus was discovered to have B/T MPAL on bone marrow biopsy during hospitalization for dyspnea due to pulmonary embolism. The patient achieved brief remission with blinatumomab treatment before succumbing to neutropenic sepsis. The lack of sufficient data to guide therapy in MPAL remains a challenge, highlighting the potential of new targeted approaches such as blinatumomab to improve outcomes in relapsed/refractory MPAL.

## Introduction

Mixed phenotype acute leukemia (MPAL) accounts for approximately 2-5% of acute leukemia cases [1,2]. The disease is characterized by the presence of immature cells displaying immunophenotypic features of more than one lineage. MPAL most commonly presents as a combination of T/myeloid markers or B/myeloid markers [3] while the B/T lymphoid subtype is one of the rarest variants, accounting for less than 5% of all MPAL cases [4].

Due to the rarity of the disease and its subtypes, there is a scarcity of well-studied therapeutic options for MPAL. Treatment is typically based on case series and small retrospective studies that utilize established regimens for acute lymphoblastic leukemia (ALL). However, the overall 5-year survival rate remains low at around 20% [5]. Moreover, there is a lack of data to guide treatment for patients with relapsed/refractory (R/R) MPAL [6].

In 2018, blinatumomab became the first FDA-approved bispecific T cell engager (BiTE) for the treatment of R/R B-ALL. Blinatumomab is a novel monoclonal antibody treatment that targets CD19 and CD3, improving outcomes in patients with R/R B-ALL [7,8]. While there have been reports of the successful use of blinatumomab in B/myeloid MPAL [9], no cases have been reported of its use in B/T lymphoid MPAL. In this study, we present a patient with R/R B/T lymphoid MPAL who received blinatumomab monotherapy.

## Case presentation

A 64-year-old man was hospitalized in August 2020 with dyspnea and was diagnosed with deep vein thrombosis and pulmonary embolism. A routine complete blood count revealed a white blood cell count of 12,100/cu.mm, with a predominant lymphocytosis (68%), a hemoglobin level of 8.5 g/dL, and a platelet count of 113,000/uL. Peripheral smear examination showed atypical, large cells with immature features and prominent nucleoli. The patient had a medical history of bladder cancer treated surgically in 2011, resulting in a urostomy and ileostomy, stage 3 chronic kidney disease, and type 2 diabetes mellitus.

**Figure 1:**
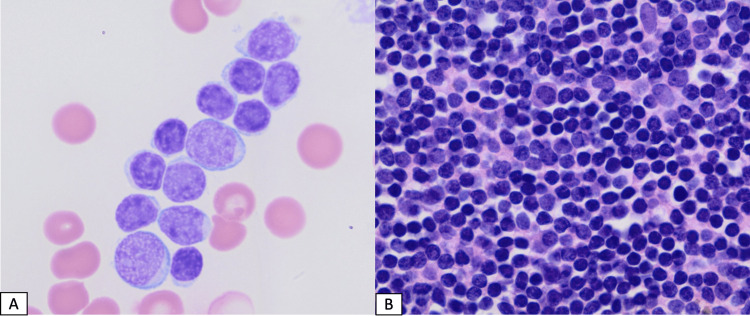
Bone marrow aspiration (A) Bone marrow aspirate shows blasts that are variable in size with a high nucleus-to-cytoplasmic ratio, dispersed nuclear chromatin, and prominent nucleoli (Wright’s, 100X), and (B) bone marrow biopsy shows sheets of lymphoid blasts (H&E, 1000X)

**Figure 2:**
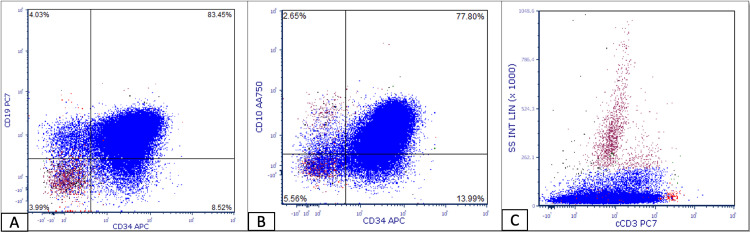
Flow cytometry Flow cytometry plot shows the blast population (blue color) post for CD34 expressing (A) CD19, (B) CD10, and (C) cytoplasmic CD3 consistent with B/T-lymphoid mixed phenotype acute leukemia

Bone marrow biopsy and aspirate showed 100% marrow cellularity with sheets of blasts (Figure 1A-1B). Flow cytometry showed blasts expressing TdT with B-cell markers including CD19, CD10, and CD79a and T-cell markers including cCD3, CD2, CD5, CD10, CD7 (Figure 2A-2C). Myeloid markers CD13 and CD33 were dim, and MPO and CD117 were negative, therefore not meeting the European group for immunological characterization of acute leukemias (EGIL) criteria (score of >2 for myeloid lineage) for B/myeloid biphenotypic classification. Fluorescence in situ hybridization (FISH) was negative for Philadelphia chromosome (Ph) ABL1/BCR gene fusion t(9;22). Karyotype was normal; 46, XY. Lumbar puncture showed no CNS leukemia. Based on the patient's immunophenotype and cytogenetic information, the diagnostic criteria for B/T lymphoid MPAL per the 2016 World Health Organization (WHO) criteria were met.

 

The patient received induction therapy with hyperfractionated cyclophosphamide, vincristine, doxorubicin, and prednisolone (hyper-CVAD)/high‐dose methotrexate and cytarabine (MA). During the post-induction period, he experienced febrile neutropenia and mild disseminated intravascular coagulation (DIC). A repeat bone marrow biopsy after two cycles of chemotherapy showed minimal disease with less than 1% abnormal blast population expressing B- and T-cell markers.

He completed eight cycles of hyper-CVAD/MA treatment with minimal toxicities. A bone marrow biopsy after completing chemotherapy revealed an aberrant blast population (10%) consistent with (R/R) leukemia. Compared to the initial flow cytometry results, the B-lineage marker CD19 was expressed, but there was a loss of CD10 and CD79a expression. The T-lineage marker cytoplasmic CD3 remained present.

Treatment with blinatumomab was initiated at a dose of 9 mcg/24hr intravenous continuous infusion on days one through seven, which was then increased to 28 mcg/24hr for the remainder of the 28-day infusion period. The patient experienced grade two neurotoxicity with mild confusion that resolved with dexamethasone. A bone marrow biopsy after completing the first cycle of blinatumomab showed a hypocellular marrow with 10% cellularity and no evidence of minimal residual disease on flow cytometry. Evaluation for an allogeneic bone marrow transplant was conducted during this time, but the patient declined to proceed with the transplant. He completed three cycles of blinatumomab on a 42-day schedule with minimal side effects. However, the patient decided to discontinue therapy at that point due to logistical constraints preventing him from being able to make the frequent follow-up appointments required. One month later, he experienced disease relapse with T-cell acute leukemia and complete loss of B-cell markers, including CD19. He passed away shortly thereafter due to neutropenic sepsis.

## Discussion

MPAL is a rare subtype of acute leukemia [10]. Two classification approaches, the EGIL and the WHO classification of myeloid neoplasms and acute leukemia, are used for MPAL classification [11]. Among the sub-classifications of MPAL, B/T lymphoid MPAL is the rarest form. The disease primarily affects males and has a bimodal age distribution, with the highest incidence in patients younger than 19 and older than 60. While there is no significant difference in the incidence of MPAL among ethnicities, black patients have a statistically higher risk of death from MPAL compared to their white counterparts [10]. Overall, the risk of death from MPAL is estimated to be 60% higher than in ALL and 25% higher than in acute myeloid leukemia (AML) [12].

Initial treatment often involves ALL-type regimens. A review of 100 patients with MPAL who received ALL-type therapy showed a median overall survival (OS) of 18 months, with a median survival of 139 months for children versus 11 months for adults (P < 0.001), indicating a worse prognosis in adults [10]. Studies have shown mixed results when attempting to determine outcome differences between AML and ALL induction treatments [13]. Post-remission allogeneic hematopoietic cell transplantation appears to improve outcomes compared to chemotherapy alone [2,14,15].

R/R MPAL is generally associated with a poor prognosis, and there is no consensus on optimal treatment. AML-type salvage therapy is often used, but targeted therapies are emerging as a potential approach. Few case reports have described patients with B/myeloid MPAL treated with blinatumomab, resulting in complete remission [9,16]. This case report provides further evidence of the potential activity of CD19-targeted therapy (blinatumomab) for MPAL, regardless of immunophenotypic aberrancy associated with CD19 expression on leukemic blasts. It also supports the use of blinatumomab as a bridge to allogeneic hematopoietic cell transplantation. Several ongoing clinical trials are investigating the use of blinatumomab in MPAL, but no results have been published to date [17].

## Conclusions

The lack of adequate data to guide therapy in MPAL remains a challenge. Prospective clinical trials and large-scale retrospective studies are needed to determine optimal treatment regimens for MPAL. Although there have been few reported cases of MPAL treated with blinatumomab, this case represents the first documented treatment of B/T lymphoid MPAL with blinatumomab monotherapy. Given the high relapse rate with chemotherapy, new targeted approaches, such as blinatumomab, may improve outcomes in R/R MPAL.
